# Feasibility Study of Anaerobic Baffled Reactor Coupled with Anaerobic Filter Followed by Membrane Filtration for Wastewater Treatment

**DOI:** 10.3390/membranes13010079

**Published:** 2023-01-08

**Authors:** Aamir Khan, Sher Jamal Khan, Waheed Miran, Waqas Qamar Zaman, Alia Aslam, Hafiz Muhammad Aamir Shahzad

**Affiliations:** 1 Institute of Environmental Sciences and Engineering, School of Civil and Environmental Engineering, National University of Sciences and Technology (NUST), Sector H-12, Islamabad 44000, Pakistan; 2 School of Chemical and Materials Engineering, National University of Sciences and Technology (NUST), Sector H-12, Islamabad 44000, Pakistan

**Keywords:** anaerobic baffled reactor, anaerobic filter, Decentralized Wastewater Treatment System (DWTS), woven fiber microfiltration

## Abstract

The performance of a Decentralized Wastewater Treatment System (DWTS) comprising an Anaerobic Baffled Reactor (ABR) and an Anaerobic Filter (AF) and Membrane Filtration (MF) module was studied for domestic wastewater treatment. The efficiency of the system was evaluated by running ABR at four different HRTs (14, 12, 10, and 8 h) resulting in COD removal efficiencies of 74, 72, 69, and 65%, respectively. The performance of AF using four different filtration media, i.e., PVC pipe (25 mm), PVC pipe (20 mm), PVC pipe (15 mm), and Kaldnes K3, was determined at optimized HRT (12 h). Among all the filtration media tested, the highest performance efficiency of the system was found with the PVC pipe (20 mm), which showed COD, TP, and TKN removal of 79, 32, and 63%, respectively. The efficacy of the system was proven via significant COD and turbidity removal of 94.6 and 87.2%, respectively, by the combined system.

## 1. Introduction

The increase in population along with the reliance of industries, particularly the textile sector, on freshwater resources in recent years has led to severe deterioration of available water quality [1]. Approximately 80% of wastewater is directly discharged into surface water bodies without any treatment, which imposes significant stress on water availability [2]. In order to cope with freshwater shortage, wastewater treatment and reclamation seem to be the only sustainable solution [3]. Valuable soil supplements and clean energy (biogas) can be harnessed from wastewater treatment in addition to providing clean water. The concept of re-using wastewater entails the requirement to develop efficient, economically viable, and environmentally friendly wastewater treatment technologies, which presents an obstacle in research and innovation in developing countries, often leading to the selection of inappropriate technology. Various treatment technologies for wastewater reclamation have been in place for centuries and include physical, chemical, and biological treatment options. Among different treatment technologies, the focus is given to decentralized treatment systems as compared to centralized wastewater treatment systems owing to their ease of operation and maintenance, especially in developing countries. Moreover, the cost of extended wastewater collection/transmission and skilled labor also makes the decentralized option the viable choice.

Biological treatment systems offer substantial benefits over other treatment options [4]. However, the application of aerobic wastewater treatment is obstructed due to the huge energy requirements for aeration. Moreover, such processes produce excess sludge, which is another major problem that needs to be addressed [5]. Hence, on the other hand, anaerobic wastewater treatment technology has the advantage of reducing the organic load of wastewater and recovering energy through biogas generation [6]. Several anaerobic systems available for wastewater treatment include the Up-flow Anaerobic Sludge Blanket Reactor (UASB), the Anaerobic Continuously Stirred Reactor, the Anaerobic Baffled Reactor (ABR), etc. Among these, the ABR is the preferred choice, owing to its longer biomass retention time, better resilience towards organic shock loadings, and special ability to partly separate the various phases of anaerobic catabolism [7].

ABRs are currently seen as a sustainable alternative to conventional anaerobic wastewater treatment options for practical applications. The ABR system consists of a series of anaerobic reactors in which the wastewater flows through different compartments separated by baffles [7]. The series of compartments in ABR enable increased contact time between the wastewater and the active biomass accumulated in the system [8]. ABRs can naturally segregate the phases of hydrolysis, acidogenesis, and methanogenesis among the sequential compartments of the reactor and improve the process of methane generation [9]. 

Recently, they have been studied for the treatment of various wastewaters including dyeing, dormitory, and fish processing wastewaters [10,11]. Recent research on ABRs practicability has particularly focused on nitrogen removal resulting from the seasonal variations (low-temperature conditions) and the reactor’s design considerations (baffles configurations) while maximizing the performance efficiency of wastewater treatment [12]. The previous studies on wastewater treatment have verified the robustness of the system for producing higher-quality effluents under varying HRTs, temperatures, and system configurations [10,11,12].

However, the widespread application of this technology is still hindered owing to certain factors, including but not limited to inferior-quality effluent compared to the conventional activated sludge process in terms of total suspended solids (TSS), nutrients, and pathogen removal [13]. Hence, the effluents from ABR need further treatment to remove suspended solids and reduce the nutrient load in an attached growth process, such as an anaerobic filter. The anaerobic filter system uses up-flow bioreactors that are filled with support media. The packing material is the same as that used in aerobic attached growth processes. The specific surface area usually ranges from 100–1000 m^2^/m^3^ depending on the media, with a void volume of >85% [12]. The existence of packing media allows the growth of attached biomass, but the principal role of the media is to retain suspended growth. 

Further post treatment of the resulting effluent is needed to meet the wastewater reuse standards. Membrane filtration as a final polishing technique offers a practicable solution for reusing treated wastewater for various purposes such as agriculture, landscaping, etc. [14].

Though several studies have evaluated the performance of ABRs for wastewater treatment, the application of a lab-scale ABR unit as a pre-study of conventional full-scale treatment under a temperate climate has been limited. A very limited number of studies have evaluated the post-treatment systems to further harness the potential of wastewater for reuse. In this research, ABR was coupled with AF for biological degradation using different media to evaluate the extended treatment performance through suspended and attached growth processes. The integrated system was further coupled with a flat-sheet woven fiber microfiltration membrane with a pore size of 1–3 µm to evaluate the water reuse potential.

## 2. Materials and Methods

### 2.1. Reactor Design

**Figure 1:**
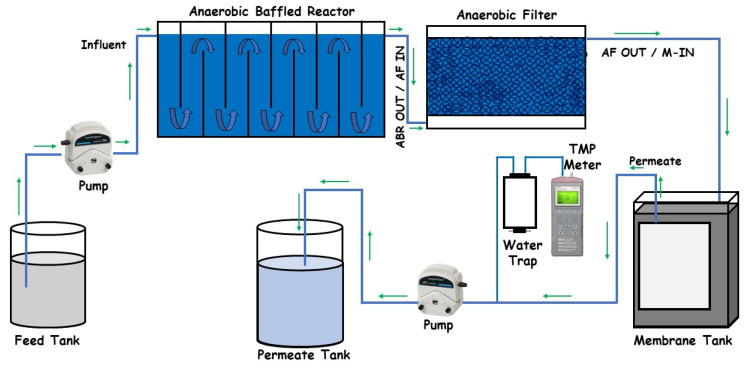
Process flow diagram of decentralized wastewater treatment system.

The process flow diagram of the whole Decentralized Wastewater Treatment System used for this study is given in Figure 1. A lab-scale anaerobic baffled reactor (ABR) was fabricated from an acrylic sheet with a 6 mm thickness and an effective volume of 21 L. The reactor consisted of six chambers, and on top of each chamber, a gas outlet with a 6 mm diameter valve was provided. Sampling ports (6 mm diameter valves) in each chamber of the ABR were also installed to check the performance of individual compartments. 

The anaerobic filter (AF) used in the study was also fabricated from an acrylic sheet with a 6 mm thickness. The total depth of the system was 35.5 cm (14 inches) with a media depth of 30.5 cm (12 inches). To prevent the media from fluidization, a mesh with a pore size of 12 mm was installed at the bottom of the media bed and 2.54 cm above the AF reactor bed. Similarly, another mesh with a pore size of 6 mm was installed at the top of the media bed and just below the effluent line, which prevented sludge from escaping into the effluent line. 

A 5 L tank fabricated from an acrylic sheet was used for membrane filtration. Water was fed into the tank from the bottom and an effluent port was provided 2.54 cm below the top for excess water discharge or as an overflow line. The membrane module fabricated from High-Density Polyethylene (HDPE) material, with a 1 cm thickness, a 15 cm length, and an 18 cm width, was placed in this reactor. Two ports were provided at the top of the module for the collection of permeate. The membrane used was a flat sheet Woven Fiber Micro Filtration (WFMF) membrane with a pore size of 1–3 μm. The surface area of the membrane was 0.044 m^2^ while operational flux was maintained at 6 LMH with the help of a peristaltic pump.

### 2.2. Filter Media Characteristics

The media used in the study was the conventional Kaldnes K3 media and locally available PVC corrugated tubes with a diameter of 15 mm. The PVC media was cut into pieces of different lengths, i.e., 25 mm, 20 mm, and 15 mm, to study the performance of AF (detailed specifications mentioned in Table 1). All these media were filled in individual filters, and the performance of all the media was compared, i.e., Kaldnes K3, PVC 25 mm, PVC 20 mm, and PVC 15 mm. The approximate surface areas of the cylindrical media used were calculated using Equation (1).

(1) $$A=2\pi rh+2\pi r^{2}$$

### 2.3. Seed Sludge and Wastewater Characteristics

Seed sludge used for start-up of the reactor was collected from a lab-scale anaerobic CSTR treating municipal wastewater. Each compartment of the ABR was fed with approximately 10% volume of the seed sludge. The anaerobic sludge had ORP, pH, and TS values of −340 mV, 6.9, and 4.8 ± 0.1%, respectively.

Synthetic wastewater was prepared on a daily basis and the composition of the wastewater is reflected in Table 2.

### 2.4. Operational Strategy

This research was carried out in three different phases, namely, the optimization of HRT of ABR, performance evaluation of AF media, and polishing via membrane filtration. During all phases, the anaerobic conditions of the system were ensured through regular monitoring of pH, ORP, and the VFA/Alkalinity ratio.

In phase I, the ABR was run at four different HRTs of 14, 12, 10, and 8 h to optimize HRT following a few weeks for acclimatization. In phase II, AF was run at a fixed HRT of 12 h (optimized HRT in ABR). The ABR effluent was carried over to the anaerobic filter to study the performance of different media, with each media having a unique packing density. In phase III, the effluent from the AF reactor was fed into the membrane tank for final polishing to meet wastewater reuse standards. 

To measure the intrinsic membrane resistance (R_m_), cake layer resistance (R_c_), and pore resistance (R_p_), the resistance test was used. The total resistance (R_t_) was measured by the equation from Darcy’s law:

R_t_ = ΔP/J·µ·f_t_

where:

R_t_ = R_c_+ R_p_+ R_m_

It is to be noted that R_t,_ R_c_, R_p_, and R_m_ (1/m) represent the total hydraulic resistance, cake layer resistance, pore blockage resistance, and intrinsic membrane resistance, respectively. The cake layer resistance is primarily caused by the deposition of sludge flocs and colloids, while the pore resistance is offered, resulting from the blockage of membrane pores by dissolved matter. J is the permeate flux, ΔP is the trans-membrane pressure, µ is the dynamic viscosity of water, and f_t_ = e^−0.0239(T − 20)^ is the temperature correction factor [15]. Total hydraulic resistance (R_t_) was measured using the data of TMP obtained for the membrane. R_p_ was calculated by removing the sludge cake from the membrane surface and measuring the resistance while filtering distilled water, while R_m_ was measured by filtering distilled water after chemical cleaning of the membrane. 

### 2.5. Analytical Methods

Chemical oxygen demand (COD), Total Kjeldahl Nitrogen (TKN), Phosphorus (PO_4_^3−^-P), VFA/alkalinity, ORP, pH, and biogas are the parameters that were monitored to check the performance of the integrated system. COD, TKN, and PO_4_^3−^-P were measured using standard methods [16]. VFAs and alkalinity were determined using the titration method [16] with an endpoint pH of 4.5. ORP and pH were measured using a portable meter (Hanna Instruments Ltd., HI 83141, Bedfordshire, UK) with an ORP probe (HI 3131B ORP electrode) and pH probe (HI 1230B pH electrode). 

## 3. Results and Discussion

### 3.1. COD and Nutrients Removal in Phase I

The most important component of wastewater treatment is COD removal to reduce the organic load for reuse or discharge, making it compliant with the applicable reuse or discharge standards. Anaerobic processes usually have better COD removal efficiency compared to aerobic processes if medium- to high-strength wastewater is considered because of longer HRTs. In Phase I, the average COD removal in ABR was observed to be 74 (±0.97)%, 72 (±1.58)%, 69 (±1.34)%, and 65 (±1.73)% at HRTs of 14, 12, 10, and 8 h corresponding to organic loading rates (OLRs) of 0.90, 1.05, 1.26, and 1.58 kg COD/m^3^ day, respectively, which affirmed the reliability of the system at each operating HRT. The results of Phase I are shown in Table 3. The system achieved a steady state shortly after the variation of each HRT, which may be associated with the uniform characteristics of wastewater, the inoculum source, and the ABR configuration. Generally, the trend of COD removal was observed to decrease with an increasing organic loading rate. Reduced efficiency of ABR was observed at shorter HRTs owing to increased organic loading, which influenced the activity of biomass. The fluctuations in COD removal efficiency were observed to be smooth at HRTs of 14 and 12 h. Primarily, the observed COD removal in ABR was associated with the anaerobic degradation of wastewater. The phosphorus removal in ABR at different HRTs of 14, 12, 10, and 8 h is also shown in Table 3. TP removal increased with an increasing HRT of the system. A similar trend was also explained by Hahn and Figueroa [17] who achieved 65% TP removal at an HRT of 15 days. The performance of ABR for COD, TKN, and TP removal did not vary considerably at HRTs of 14 and 12 h due to the sufficient activity of microorganisms as depicted in the results. 

The operation of the system clearly illustrated consistency in treatment performance under optimized conditions. Hence, the optimal operating HRT of the ABR system was found to be 12 h keeping in view the large capital requirement required at higher HRTs. Moreover, a minimum HRT of 12 h was recommended in another study by Hahn and Figueroa [17] to ensure reasonable biodegradability of particulate COD. The ABR appeared to be an appealing primary treatment to evaluate the suitability of system replication for full-scale applications, particularly in temperate climates.

### 3.2. COD Removal in Phase II

**Figure 2:**
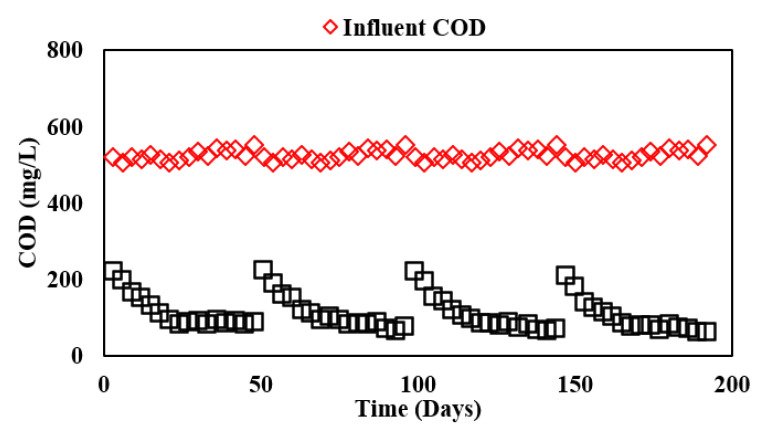
Influent and effluent COD concentrations in ABR-AF system during Phase II.

In Phase II of the study, ABR was coupled with AF to evaluate the treatment performance of various filter media for which the detailed results are depicted in Table 4 and Figure 2. The integrated system showed significant treatment performance with average COD removal of 78% with the PVC-25 filter, 79% with PVC-20 and 79% with PVC-15 filters, and 81% with the Kaldnes filter. The final effluent from AF had a COD value of less than 100 mg/L. The observed results were comparable to the findings of Nguyen, Watari, Hatamoto, Sutani, Setiadi, and Yamaguchi [11] who reported 78.3% removal of COD while treating synthetic dyeing wastewater at 23.2 h in an ABR down-flow hanging sponge (DHS) system.

The increasing trend of COD removal in different filters was observed because of the larger surface area available for biomass growth, which subsequently enhanced the treatment efficiency. The comparison of available surface area for attached growth was already discussed in Table 1. Moreover, the pH of the system ranged between 6.00 and 8.00 and the ORP was well below −250 mV, which ensured suitable ecological conditions for anaerobic microorganisms. The results of the present study were comparable to the findings of Majid and Mahna [18] in which a total COD removal of 87% was observed at an HRT of 12 h in a lab-scale moving-bed biofilm reactor (MBBR) using Kaldnes K3 media for synthetic wastewater treatment. A similar trend of COD removal using Kaldnes K3 media was also observed by Jaafari, et al. [19] where a total removal of 95.5% was observed in a modified phoredox reactor for municipal wastewater treatment at an HRT of 8 h. Shebl, Hassan, Salama, El-Aziz, and Abd Elwahed [4] also observed similar trends of COD removal (75%) in their study for treating medium-high-strength wastewater at an HRT of 24 h in ABR. Furthermore, another study on an ABR-AF system by Renuka, et al. [20] reported COD removal of 90% at an HRT of 40 h. 

Hence, in terms of process performance and effluent quality, a reasonable choice can be made between conventional Kaldnes K3 media and PVC media, which were found to exhibit the optimum efficiency for average COD removal. Moreover, among different sizes of PVC, a 20 mm length is recommended as the optimum choice from the perspective of mechanical integrity and economic viability for practical applications. A further reduction in media size may lead to media structure distortion in larger-scale reactors. 

### 3.3. TP Removal in Phase II

Phosphorus removal in anaerobic reactors is usually less compared to aerobic reactors. The removal of phosphorus in biological processes is through Phosphorus Accumulating Organisms (PAOs), which use phosphorus as the growth material [21]. 

**Figure 3:**
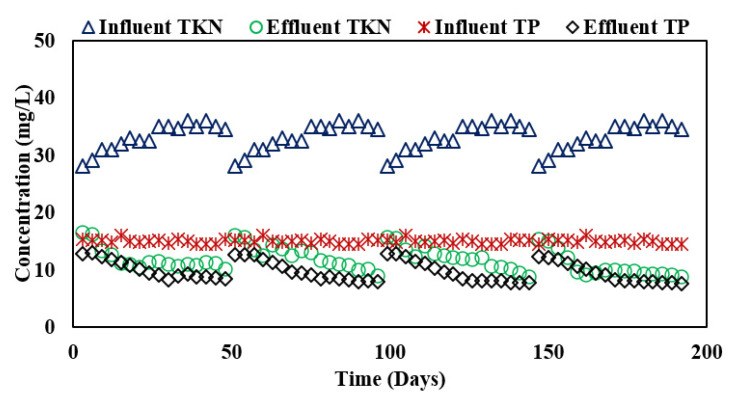
Influent and effluent TKN and TP concentrations in ABR-AF system during Phase II.

TP removal in the integrated system was found to increase in the anaerobic filter owing to greater biomass concentration in the biofilms as shown in Table 4 and Figure 3. The overall TP removal was found to be 29, 32, and 34.3% in PVC-25, PVC-20, and PVC-15 filters, respectively, while it was observed to be 35% using Kaldnes media in the filter. Shebl, Hassan, Salama, El-Aziz, and Abd Elwahed [4] also reported a similar trend of TP removal of 32% at an HRT of 48 h in ABR alone. Similarly, the reported results of TP removal were almost the same (22.5%) as observed in an ABR treating wastewater containing oxytetracycline (OTC) at 35 °C [10] and those reported by Gholipour and Stefanakis [10] for the treatment of university dormitory wastewater in ABR and hybrid constructed wetland. In the present study, a suspended growth system was coupled to an attached growth system leading to enhanced removal efficiency, which resulted in an average effluent TP concentration of 9.7 mg/L.

The observed phosphors removal occurred due to the biomass formation of the microbes. This biomass formation in anaerobic reactors is less due to longer SRTs, and thus the removal efficiency is less compared to aerobic reactors in which there is more biomass formation and sludge wastage, hence exhibiting higher TP removal efficiencies of greater than 95%. Phosphorus removal in anaerobic filters is mediated by the biofilms present and the corresponding microbial consortia, primarily the phosphorus-accumulating organisms. Such removal of phosphorus is associated with normal-temperature anaerobic systems, and the removal usually drops with increasing loading [22]. Phosphorus removal in anaerobic systems has shown rather similar removal efficiencies, i.e., 25% ortho-phosphate removal was observed while operating an up-flow anaerobic sludge blanket reactor at an HRT of 13 h [23]. The proposed system in this study achieved the discharge limits of phosphorus after the anaerobic filter. 

### 3.4. TKN Removal in Phase II

Nitrogen is a nutrient used by microbes for growth, and anaerobic treatment processes are characterized as less effective for its removal compared to aerobic processes, which are reported to have total nitrogen removal of 90% [1]. A summary of the results of the integrated ABR and AF system is reported in Table 4 and Figure 3. In this study, TKN removal, similar to TP removal, was observed to be dependent on the HRT and biomass in the integrated system. The average removal of TKN in the integrated system was observed to be 65% in PVC-25, 63% in PVC-20, 63.1% in the PVC-15 filter, and 69% in Kaldnes media resulting in effluent concentrations of 10.1, 9.9, 9.5, and 9.4 mg/L. It is evident from these results that the coupled growth processes show more nitrogen removal compared to suspended growth alone as the treatment performance is linked to the larger surface area in the attached growth process due to more bio-accumulation of nitrogen in attached biomass [24]. 

Using the processes of nitrification and denitrification for treating wastewater with high levels of ammonium-nitrogen usually requires rather high energy input. In addition, due to the presence of high ammonium and organic carbon, nitrogen removal via normal denitrification is not enough to meet regulatory standards. Pre-denitrification in such cases prior to nitrification provides a solution to decrease sludge production and results in lower operating costs. Pre-denitrification has been proven to be the most efficient strategy for the simultaneous removal of organic matter and nitrogen from wastewater. Such a process can remove up to 69% of the nitrogen in an anaerobic digestor [25].

Similar results of nitrogen removal were observed for the treatment of municipal wastewater in ABR at HRTs of 24, 18, 12, and 8 h, which achieved nitrogen removal of 49, 27, 21.6, and 14%, respectively [26]. In addition, Zha, et al. [27] also observed in their study a total TKN removal of 29% in ABR at an optimized HRT of 48 h, whereas, in the present study, a maximum of 63% removal was observed in Phase I employing the ABR alone. The increased TKN removal could be associated with greater bioaccumulation of nitrogen in sludge, thereby resulting in more TKN removal efficiency as compared to other reported studies. Another study conducted in 2020 reported TKN removal of 64% utilizing an anaerobic sludge blanket reactor for municipal wastewater [28]. Anaerobic processes mainly convert organic nitrogen into ammonium nitrogen, but the remaining conversion of ammonium is not as efficient because of the lack of processes such as nitrification and denitrification. In such systems, nitrogen is primarily removed via pre-denitrification and biomass accumulation [25]. The system studied in this study brought the TKN to safe discharge limits, hence there was no need to install an aerobic unit for the further reduction of TKN levels.

### 3.5. COD and Turbidity Removal in Membrane Reactor (Phase III)

The system required a final polishing technique and evaluation in order to have the necessary stable effluent quality. Therefore, in Phase III, the effluent from the integrated ABR-AF was fed into the membrane reactor in which a microfiltration woven fiber membrane was placed, and the system was run for 67 days. COD and turbidity removal efficiencies of the whole system were evaluated to establish the reliable effluent polishing option to serve the ultimate end of effluent reuse. 

**Figure 4:**
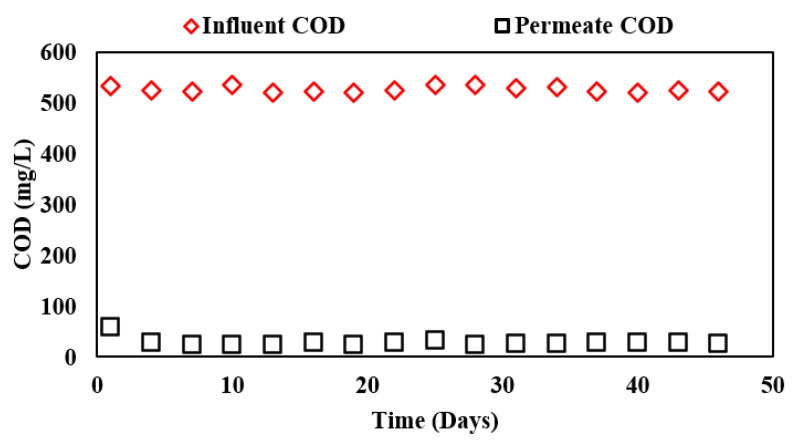
Influent and effluent COD concentrations in ABR-AF coupled membrane system during Phase III.

COD removal of the membrane reactor is shown in Figure 4. The line with red markers represents influent COD in the system whereas the line with black markers represents the permeate COD from the complete ABR-AF coupled membrane system.

It can be observed that the combined system achieved COD removal of 94.6% with an effluent COD concentration of approximately 28.1 mg/L at an HRT of 24 h for the biological system at a membrane flux of 6 LMH. The observed results were in accordance with another study employing a hollow-fiber membrane with a pore size of 0.4 microns used for polishing the effluent, which reported a COD removal of 96% at an operational flux of 30 LMH at a combined HRT of 3 h [8]. Likewise, COD removal was further verified by Sung, Katsou, Statiris, Anguilanom and Malamis [9] who reported 93.3% removal using the integrated ABR-MBR process at a membrane flux of 20 LMH for municipal wastewater treatment. Although a low operational flux of 6 LMH in this study is considerably less than the conventional flux of 15–30 LMH for hollow-fiber or flat-sheet membranes, it can be useful for low-cost polishing systems. 

**Figure 5:**
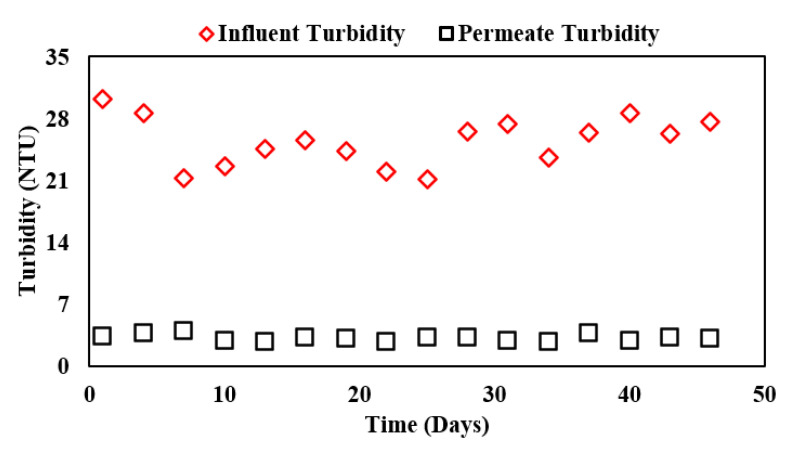
Influent and effluent turbidity concentrations in ABR-AF coupled membrane system during Phase III.

The suspended solids or turbidity removal of the overall system is reflected in Figure 5. The line with red markers shows the influent turbidity in the integrated system whereas the line with black markers shows the permeate turbidity from the complete ABR-AF coupled membrane system. It was inferred that the MF membrane with 1–3 μm pore size can effectively remove almost all the suspended solids present in the receiving wastewater.

The steady removal efficiency was observed to be 87.2% with minimum effluent turbidity of 2.8 and an average of 3.2 NTU, which is in line with the suspended solids removal efficiency of 99% reported by Ratanatamskul, Charoenphol, and Yamamoto [8] using a 0.4 μm hollow-fiber membrane. 

### 3.6. TMP Profile of the Flat-Sheet Membrane Module

**Figure 6:**
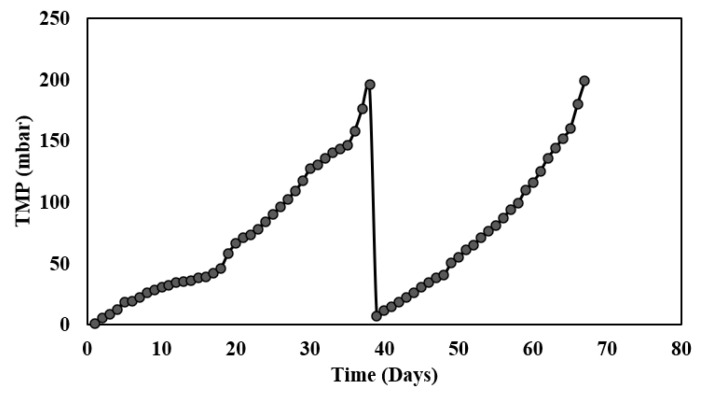
TMP profile of membrane during Phase III.

The complete ABR-AF coupled membrane system was run for approximately 67 days, and the fouling behavior was observed through a data logging manometer. The plot of the recorded TMP is shown in Figure 6. It is evident from the figure that a slow TMP increase over a longer period was observed, leading to longer membrane runs. Thus, the membrane performed very well in removing the suspended solids without much of an increase in TMP. On the 35th day of membrane operation, the TMP began to rise abruptly, thus indicating a TMP jump. The membrane achieved the threshold (200 mbar) within 2 days of the start of the TMP jump and fouled on the 37th day of the run, which indicated an MF membrane with a 1–3 μm pore size can produce better results with less fouling rates compared to a 0.4 μm membrane. The membrane was taken out of the reactor for recovery cleaning prior to the beginning of the second run, and the operating flux was restored after the cleaning. Hence, the total membrane resistance (R_t_) was evaluated to be 6.73 × 10^12^ 1/m during the study period. Following chemical cleaning, resistance reasonably decreased to 0.1 × 10^12^ 1/m (R_m_). The membrane operated for almost 30 days in the second run.

## 4. Conclusions

The lab-scale ABR-AF operating at a 12 h HRT was evaluated for wastewater treatment, which gave significant COD removal of 78–81% throughout the operational phases with all tested filter media. There was a slight difference in the average COD removal efficiency of Kaldnes media (81%) and PVC 20 mm media (79%). The conventional Kaldnes media can thus be easily replaced by locally available low-cost PVC media. 

The integrated system (ABR-AF) with a total HRT of 24 h thus produced water that meets the local discharge standards with an effluent COD of 113 mg/L. For agricultural reuse, membrane filtration is recommended to bring down the effluent COD to <50 mg/L and turbidity to <5 NTU. The MF membrane serves as a better option for post treatment as less fouling is incurred owing to the large pore size of the membrane as compared to UF membranes.

## Figures and Tables

**Table 1:** Surface area of filter media used in the study.

Media	Surface Area/Piece (mm^2^)	Total Pieces Filled	Total Surface Area Available (mm^2^)
Kaldnes K3 (25 mm × 10 mm)	500 mm^2^/mm^3^	687	2.45 × 10^6^
PVC 25 mm	1532	764	1.17 × 10^6^
PVC 20 mm	1296	955	1.24 × 10^6^
PVC 15 mm	1060	1273	1.35 × 10^6^

**Table 2:** Synthetic wastewater characteristics.

Compound	Formula	Concentration (mg/L)
Glucose	C_6_H_12_O_6_	520
Ammonium Chloride	NH_4_Cl	133.6
Potassium Dihydrogen Phosphate	KH_2_PO_4_	17.2
Calcium Chloride	CaCl_2_	4.8
Magnesium Sulfate	MgSO_4_	4.8
Ferric Chloride	FeCl_3_	0.5
Sodium Bicarbonate	NaHCO_3_	80
Cobalt Chloride	CoCl_2_	0.05
Zinc Chloride	ZnCl_2_	0.05
Nickel Chloride	NiCl_2_	0.05

**Table 3:** Performance of ABR system during Phase I.

ABR System				
HRT (h)	14	12	10	8
OLR (kgCOD/m^3^.Day)	0.90 (±0.02)	1.05 (±0.02)	1.26 (±0.03)	1.58 (±0.04)
Effluent COD (mg/L)	125.8 (±4.88)	147.7 (±7.86)	166.4 (±5.19)	183.6 (±6.70)
COD Removal (%)	74 (±0.97)	72 (±1.58)	69 (±1.34)	65 (±1.73)
Effluent TKN (mg/L)	12.38 (±1.87)	15.74 (±2.89)	20.28 (±1.46)	26.12 (±1.19)
TKN Removal (%)	63 (±0.89)	56 (±1.31)	44 (±1.42)	27 (±3.34)
Effluent TP (mg/L)	9.4 (±1.63)	9.8 (±1.32)	10.05 (±1.1)	10.4 (±1.49)
TP Removal (%)	35 (±1.07)	33 (±1.98)	31 (±0.58)	28 (±0.95)

**Table 4:** Performance of integrated system (ABR + AF) during Phase II.

Media Filled	25 mm	20 mm	15 mm	Kaldnes
Number of Replicates	15	15	15	15
Operational Days	0–50	51–99	100–146	147–192
Influent COD (mg/L)	534 (±13.7)	534 (±13.7)	534 (±13.7)	534 (±13.7)
Effluent COD (mg/L)	118 (±23)	113 (±21)	112 (±25)	102 (±18)
COD Removal (%)	78 (±3)	79 (±5)	79 (±4)	81
Influent TKN (mg/L)	33.48 (±2.0)	33.48 (±2.0)	33.48 (±2.0)	33.48 (±2.0)
Effluent TKN (mg/L)	11.75 (±1.9)	12.16 (±1.7)	11.86 (±2.1)	10.42 (±2.2)
TKN Removal (%)	65 (±2.8)	63 (±3.2)	63.1 (±4.5)	69 (±1.5)
Influent TP (mg/L)	14.48 (±0.60)	14.48 (±0.60)	14.48 (±0.60)	14.48 (±0.60)
Effluent TP (mg/L)	10.16 (±1.6)	9.91 (±1.26)	9.51 (±1.52)	9.43 (±1.68)
TP Removal (%)	29 (±0.58)	32 (±1.25)	34.3 (±2.04)	35 (±1.34)
ORP (mV)	−334 (±12.8)	−259 (±56.7)	−253 (±57.5)	−342 (±11.1)
pH	7.19 (±0.13)	7.16 (±0.05)	7.16 (±0.07)	7.25 (±0.27)

## Data Availability

Not applicable.
